# Microwave-assisted synthesis and development of novel penicillinoate@copper metal-organic frameworks as a potent antibacterial agent

**DOI:** 10.3389/fchem.2023.1331933

**Published:** 2024-01-11

**Authors:** Adnan Hashim Abdulkadhim, Suhair Mohammad Husein Kamona, Haider Falih Shamikh Al-Saedi, Anmar Ghanim Taki, Abdul-Hameed. M. Hamoody, Sarah A. Hamood, Safia Obaidur Rab, Ahmed Ali Amir, Ashwaq Talib Kareem, Ahmed Alawadi, Ali Ihsan

**Affiliations:** ^1^ Department of Computer Engineering, Technical Engineering College, Al-Ayen University, Thi-Qar, Iraq; ^2^ Department of Medical Laboratories Technology, Al-Manara College for Medical Sciences, Amarah, Iraq; ^3^ Department of Pharmaceutical, Faculty of Pharmacy, University of Al-Ameed, Karbala, Iraq; ^4^ Department of Radiology and Sonar Techniques, Al-Noor University College, Nineveh, Iraq; ^5^ Department of Medical Laboratories Technology, Al-Hadi University College, Baghdad, Iraq; ^6^ Department of Medical Engineering, Al-Esraa University College, Baghdad, Iraq; ^7^ Department of Clinical Laboratory Sciences, College of Applied Medical Sciences, King Khalid University, Abha, Saudi Arabia; ^8^ Department of Medical Laboratories Technology, AL-Nisour University College, Baghdad, Iraq; ^9^ Department of Medical Engineering, College of Pharmacy, National University of Science and Technology, Dhi Qar, Iraq; ^10^ College of Technical Engineering, the Islamic University, Najaf, Iraq; ^11^ College of Technical Engineering, the Islamic University of Al Diwaniyah, Diwaniyah, Iraq; ^12^ College of Technical Engineering, the Islamic University of Babylon, Babylon, Iraq; ^13^ Department of Medical Laboratories Techniques, Imam Ja’afar Al‐Sadiq University, Al-Muthanna, Iraq

**Keywords:** microwave irradiation, penicillin, copper metal–organic framework, Gram-positive bacterial strains, Gram-negative bacterial strains

## Abstract

Recently, nanoscience, especially metal–organic frameworks (MOFs), has been used to increase the effectiveness and properties of drugs. In this study, by using microwave irradiation; penicillin, which is a known antibiotic; and copper metal–organic frameworks (Cu-MOFs), a new penicillinoate@copper metal–organic framework (penicillinoate@Cu-MOF) was synthesized. The structure and characterization of the newly synthesized compound were determined using FT-IR spectrums, EDAX analysis, elemental analysis, XRD patterns, SEM images, nitrogen adsorption/desorption curves, and TGA curve. Then, its antimicrobial effects were evaluated on numerous Gram-positive and Gram-negative bacterial strains and were compared with those of penicillin and gentamicin. In continuation of the biological activities, antioxidant tests were performed on the compounds using the DPPH method. For biological activities, the synthesized penicillinoate@Cu-MOF is much more effective than penicillin and Cu-MOF. The loading of penicillin on the nanostructure and the presence of copper in the final composition can be attributed to the high antibiotic properties of the synthesized composition.

## 1 Introduction

**FIGURE 1:**
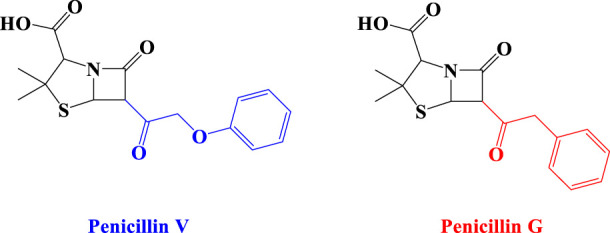
The Structure of penicillin V (for oral use) and penicillin G (for injection use).

Penicillin, available in the market in two forms, penicillin V (for oral use, Figure 1A) and penicillin G (for injection use, Figure 1B), is a potent antibiotic that is effective against a wide range of bacteria. This antibiotic was discovered in 1928 and was mass-produced and entered the market in 1946 (Miller, 2002; Lobanovska and Pilla, 2017).

**FIGURE 2:**
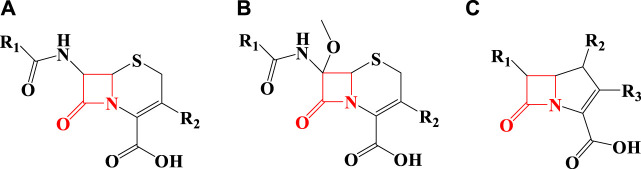
The structure of some beta-lactam antibiotics [**(A)** Cephalosporins, **(B)** Cephamycin, **(C)** Carbapenems].

As seen in Figure 1, penicillin has a beta-lactam in its structure and is included in the category of beta-lactam antibiotics. Other beta-lactam antibiotics include cephalosporins (Figure 2A), cephamycin (Figure 2B), and carbapenems (Figures 2C) (Lima et al., 2020).

Beta-lactam antibiotics destroy bacteria with the following three steps: 1) binding to the binding protein in the bacterial cell wall; 2) inhibiting transpeptidases as a result of transpeptidation, which means cross-linking in the construction of bacterial cell wall peptidoglycans; and 3) finally, autolytic enzymes called murine hydrolases are activated in bacteria that have been exposed to penicillin and cause the destruction of peptidoglycans. The result of these processes is the destruction of the bacterial wall and of the bacterial cell (Waxman and Strominger, 1983; Pandey and Cascella, 2019; Lima et al., 2020).

However, the indiscriminate use of antibiotics has led to the resistance of some bacteria to antibiotics (Bungau et al., 2021). The synthesis and reporting of new antibiotics, changes in the structure of current antibiotics, and the use of new technologies such as nanotechnology are used and have been reported for this problem (Şen Karaman et al., 2020; Okeke et al., 2022).

Nanotechnology, which has been developing recently, is used in many fields, including industry (Malik et al., 2023) and medicine (Saxena et al., 2020). Nanofibers, nanotubes, and nanowires are some examples of nanocompounds (Kargozar and Mozafari, 2018). One of the most valuable types of nanocompounds is metal–organic frameworks (Dutta et al., 2021). These compounds, mainly composed of organic linkers with metals, have many applications (Safaei et al., 2019) such as in water treatment (Yu et al., 2021), are electrically conductive (Xie et al., 2020), clean energy (Li et al., 2020), as catalysts (Kumar et al., 2021), and in photocatalysis (Liu et al., 2022). These compounds have many applications in medicine, including drug delivery, cell imaging, sensing (Safdar Ali et al., 2021), biosensors (Souza et al., 2022), and wound healing (Fardjahromi et al., 2022). There have been reports of the biological properties of these compounds, such as antitumor (Gao et al., 2021) and antimicrobial properties (Shen et al., 2020; Li et al., 2021). High porosity, specific surface area, and the type of metal used in these compounds are among the most critical factors affecting their unique properties (Razavi and Morsali, 2019; He et al., 2021). Metal–organic frameworks containing copper are an example of these compounds developed so far. There have been reports of applications such as dye removal (Firouzjaei et al., 2020), biosensing and biocatalysis (Cun et al., 2022), sensors for food safety (Cheng et al., 2021), wound dressing (Wang et al., 2020), antimicrobial activities (Abdelmoaty et al., 2022), and anti-breast carcinoma (Abu-Dief et al., 2022).

To develop and use new technologies in the medical industry and report new antibiotics with high effectiveness, in this study, penicillin was loaded onto a metal–organic framework containing copper. After identifying and confirming the structure, it was tested on a wide range of pathogenic bacteria strains. MIC, MBC, and IZD parameters were reported and compared with those of penicillin and cefazolin. In addition, the antioxidant activity on DPPH free radicals was studied. The importance of this work can be mentioned in the synthesis and confirmation of the structure of the new MOF compound containing the antibiotic penicillin and copper, which is effective against a wide range of Gram-positive and Gram-negative strains and has antioxidant activity. Even in strains with no effect of penicillin, the newly synthesized compound was effective due to the presence of the copper metal in its structure.

## 2 Materials, devices, and methods

### 2.1 Materials and devices

The chemicals and solvents used to synthesize the desired products with the highest purity were obtained from Merck and Sigma companies. Bacterial strains were prepared from the American Type Culture Collection (ATCC), and the 1 × 10^5^ CFU/mL concentration was prepared using an Agilent Cary UV-Vis 4000 spectrophotometer.

To confirm the structure and identify synthetic compounds, various analyses including FT-IR, SEM, XRD, BET, CHNS/O, and TGA were performed using Nicolet 380 FT-IR, Hitachi S-4800 FESEM, Shimadzu-6000 XRD, Horiba SA-9600 BET analyzer, Thermo EA1112 CHNS/O, and TGA Simultaneous Thermal Analyzer 6000, respectively. The corresponding images were also captured.

### 2.2 Methods

#### 2.2.1 Microwave-assisted synthesis of penicillinoate@copper metal–organic frameworks

To 25 mL of double-distilled water, 1 mmol copper (II) chloride and 2 mmol of pyridine-2,6-dicarboxylic acid were added and stirred for 15 min at 50 C to create a homogenized mixture. After cooling to room temperature, microwave radiation was irradiated at room temperature for 15 min with a power of 300 W (Ahmad et al., 2022). With nanofiltration, copper metal–organic frameworks (Cu-MOFs) were separated and washed three times with ethanol and three times with ethanol and double-distilled water. To dry the desired synthesized product, a vacuum was used for 48 h at room temperature. The synthesized Cu-MOF was identified, and the structure was confirmed by using the techniques mentioned in the Results and discussion section. It was then used to synthesize penicillinoate@copper metal–organic frameworks (penicillinoate@Cu-MOFs), and biological tests were conducted in the following steps.

To 25 mL of double-distilled water, 1 mmol of synthesized Cu-MOF and 6 mmol penicillin were added and stirred for 15 min at 50 C to obtain a homogenized mixture. After cooling to room temperature, microwave radiation was irradiated at room temperature for 15 min with a power of 300. By using nanofiltration, penicillinoate@Cu-MOF was separated and washed three times with ethanol and three times with ethanol and double-distilled water. To dry the desired synthesized product, a vacuum was used for 48 h at room temperature. The synthesized penicillinoate@Cu-M was identified, and the structure was confirmed by using the techniques mentioned in the Results and discussion section. It was then used for the biological tests conducted in the following steps.

#### 2.2.2 Antibacterial tests on penicillin and penicillinoate@Cu-MOF

In antimicrobial evaluations, the antibacterial properties of penicillin, Cu-MOF, and penicillinoate@Cu-MOF against Gram-positive and Gram-negative pathogenic bacterial strains were tested. In the tests, the IZD, MIC, and MBC were measured, and the results are provided in the Results and discussion section. For the tests, the standards, guidelines, and relevant rules of the Clinical and Laboratory Standards Institute (CLSI) were used ((Igei et al., 2016; Abdtawfeeq et al., 2022; Akhavan-Sigari et al., 2022).

At first, by using a UV-Vis device, the suspension with the concentrations of 1 × 10^5^ CFU/mL from bacterial strains in Mueller Hinton Broth (MHB) was prepared. Then, using ultrasonic dispersion, the concentrations of 1, 2, 4, … 4,096 μg/mL from Cu-MOF and penicillinoate@Cu-MOF in deionized water were prepared. The 1–4,096 μg/mL concentrations of penicillin were also prepared in deionized water.

For the MIC, 100 μL of different concentrations of compounds, 100 μL of MHB, and 10 μL of the bacterial suspension were mixed and placed in a shaker incubator at a temperature of 37 C for 48 h. Then, the lowest concentration at which turbidity was not seen was reported as the MIC. For the MBC, the MIC and five more diluted concentrations were cultured separately on Mueller Hinton Agar (MHA) and incubated for 72 h at 37 C. The concentration at which bacterial species did not grow was reported as the MBC. For IZD, the bacterial strains in MHA were cultured, and a disk blank was placed on it. Then, 10 μL of MIC was injected into the disk blanks and placed in an incubator at 37 C for 48 h. Then, by using a caliper, the created diameter was measured and reported (Igei et al., 2016; Abdtawfeeq et al., 2022; Akhavan-Sigari et al., 2022).

#### 2.2.3 Antioxidant tests on Cu-MOF and penicillinoate@Cu-MOF

To check the antioxidant activity, the 2,2-diphenyl-1-picrylhydrazyl (DPPH) method was used. First, concentrations of 25–100 μg/mL of Cu-MOF and penicillin@Cu-MOF in methanol were prepared by dispersing. Then, a concentration of 0.004% w/v of DPPH in methanol was prepared. Different concentrations of Cu-MOF/penicillinoate@Cu-MOF (1 mL each) were added to 4 mL of the DPPH solution and shaken in the dark at 25 C for 0.5 h. Then, the compounds (Cu-MOF/penicillinoate@Cu-MOF) were separated by nanofiltration, and at 517 nm, the absorbance of the sub-filter solution and DPPH solution was measured with UV-Vis (Bhaskara Reddy et al., 2015; Moghaddam-Manesh et al., 2020a). The results are provided in Section 3.

## 3 Results and discussion

### 3.1 Results of the synthesis of penicillinoate@Cu-MOF

**FIGURE 3:**
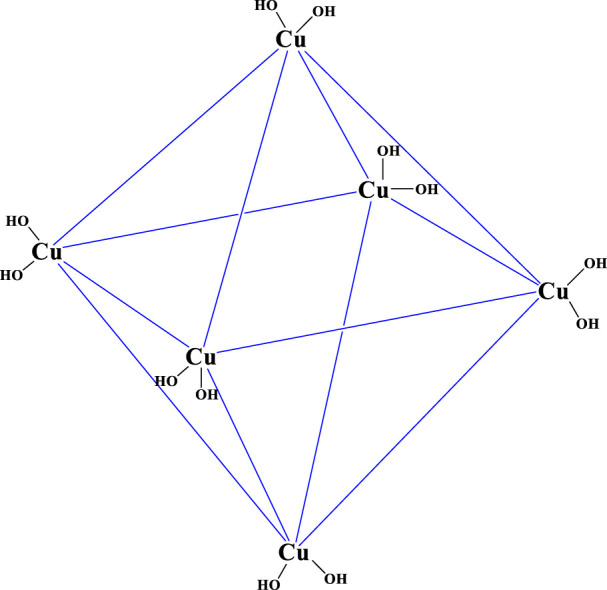
Synthesized Cu-MOF.

**FIGURE 4:**
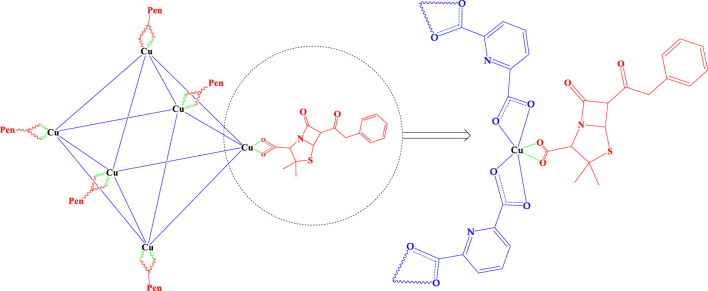
synthesized penicillinoate@Cu-MOF.

In this study, a novel penicillinoate@Cu-MOF (Figure 4) was synthesized in two steps. In the first step, under microwave conditions, using copper (II) chloride and dipicolinic acid, Cu-MOF was synthesized (Figure 3). In the second step, penicillinoate@Cu-MOF was synthesized by adding penicillin to the Cu-MOF and maintaining it under microwave conditions (Figure 4). The microwave irradiation was performed according to the conditions mentioned in Section 2–2.1. As discussed in detail below, the microwave method creates unique properties in synthetic compounds, the physical and chemical properties of which lead to increased antimicrobial properties.

FT-IR, SEM, XRD, BET, CHN, and TGA were used to confirm the structure and characteristics of the synthesized penicillinoate@Cu-MOF (Figure 4).

**FIGURE 5:**
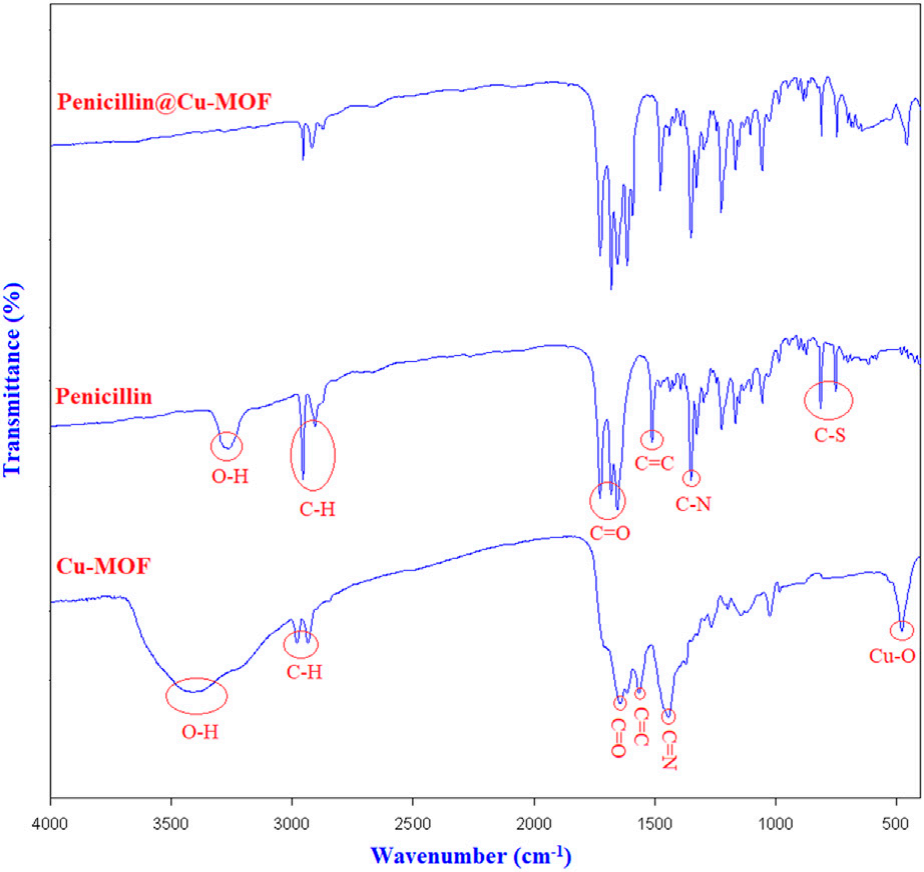
FT-IR spectrums of Cu-MOF, penicillin, and penicillinoate@Cu-MOF.

The first technique that was used to characterize and confirm the structures of Cu-MOF and penicillinoate@Cu-MOF was FT-IR spectra (Figure 5).

The FT-IR spectrum of the synthesized Cu-MOF and penicillinoate@Cu-MOF showed peaks related to Cu-O, C=N, C=C, and C-H at 508 cm^−1^ (Elango et al., 2018), 1,489 cm^−1^ (Abdtawfeeq et al., 2022), and 1,535 cm^−1^ (Asiri et al., 2022), respectively. As can be seen from the comparison in Figure 5, in the FT-IR spectrum of penicillinoate@Cu-MOF, the peaks corresponding to C-S, aliphatic ketones, and beta-lactam C=O were observed at 790 cm^−1^ (Bastian Jr and Martin, 1973; Bayati and Wei, 2014; Moghaddam-Manesh et al., 2020b), 1722 cm^−1^ (Bayati and Wei, 2014), and 1747 cm^−1^ (Thumanu et al., 2006; Esmaeilpour et al., 2016; Al-Adhami et al., 2020), respectively. Therefore, the loading of penicillin onto Cu-MOF was proven using the FT-IR spectrum.

**FIGURE 6:**
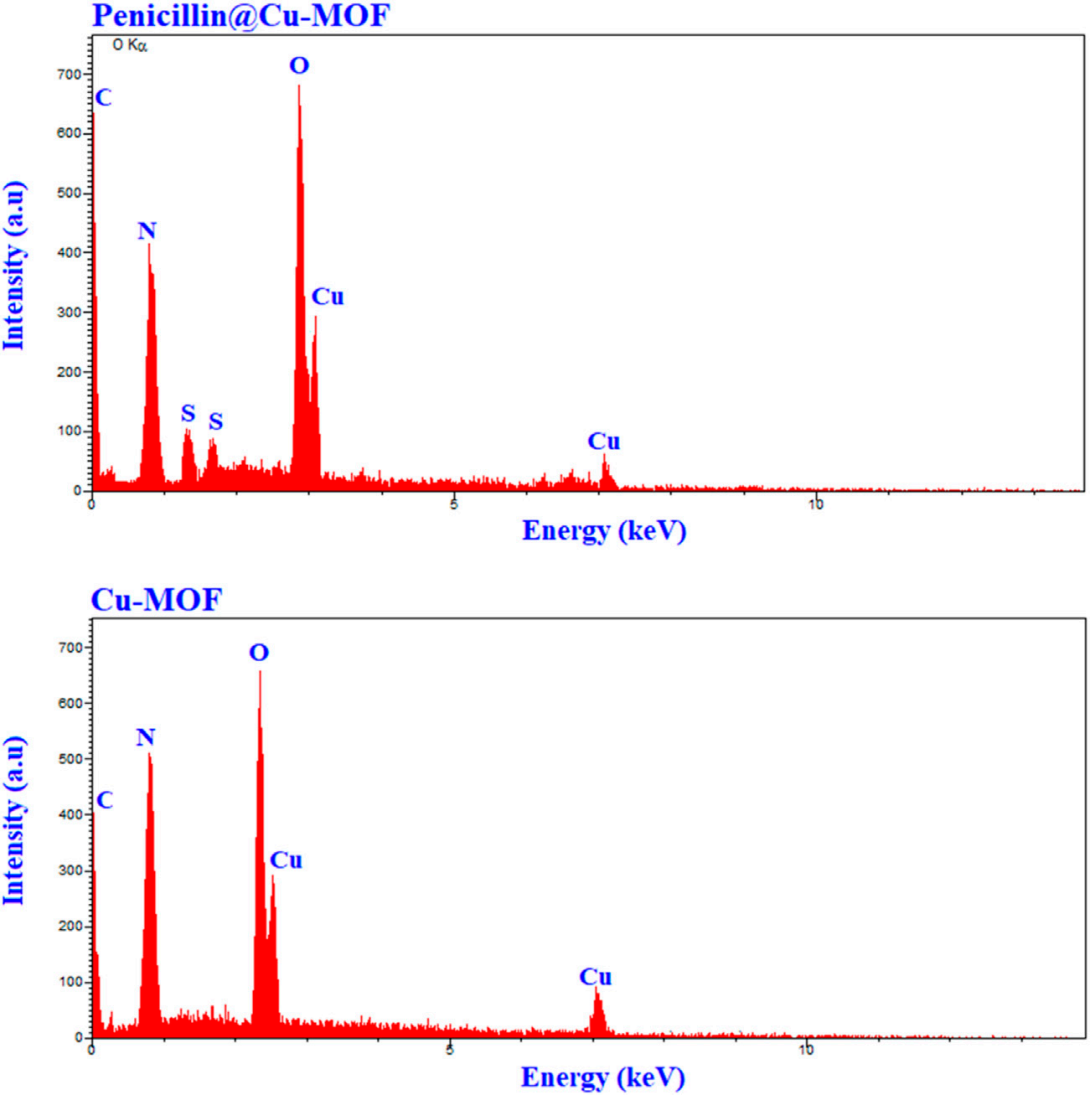
EDAX analysis of Cu-MOF and penicillinoate@Cu-MOF.

EDAX spectrums related to Cu-MOF and penicillinoate@Cu-MOF are shown in Figure 6. As can be seen, the peaks related to carbon, nitrogen, oxygen, and copper were observed in the EDAX spectrums of the Cu-MOF and the peaks related to carbon, nitrogen, oxygen, sulfur, and copper were observed in the EDAX spectrums of penicillinoate@Cu-MOF.

Comparison of the elemental analysis of Cu-MOF and penicillinoate@Cu-MOF was another technique that proved the loading of penicillin onto the Cu-MOF. The results of the elemental analysis of Cu-MOF and penicillinoate@Cu-MOF are provided in Table 1.

**TABLE 1:** Elemental analysis of Cu-MOF and penicillinoate@Cu-MOF

Element product		C	H	N	S	O	Cu
Cu-MOF	Actual	42.14	2.61	6.17	-	35.11	-
	Theoretical	42.16	2.65	6.15	-	35.10	13.94
Penicillinoate@Cu-MOF	Actual	51.95	3.57	5.67	4.36	25.98	-
	Theoretical	51.93	3.54	5.68	4.33	25.94	8.59

By comparing the results in Table 1, the increase in the percentage of carbons and the presence of sulfur based on the obtained results is another proof of the loading of penicillin on penicillinoate@Cu-MOF and, as a result, the proposed structure of Figure 3. Furthermore, the presence of penicillin in the final product, as discussed in the FT-IR and EDAX spectrums, confirms the proposed structure shown in Figure 4.

**FIGURE 7:**
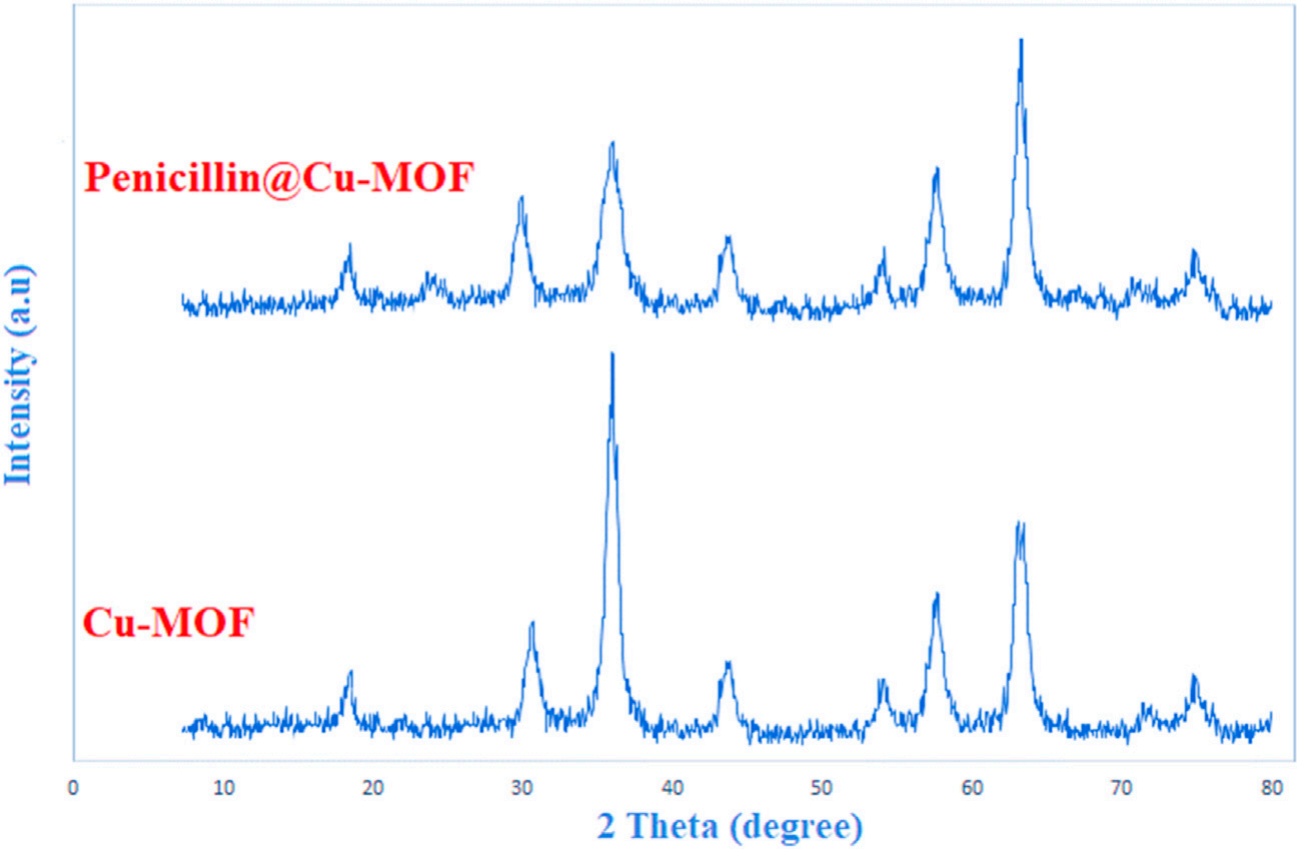
XRD patterns of Cu-MOF and penicillinoate@Cu-MOF.

The size of the synthesized Cu-MOF and penicillinoate@Cu-MOF was calculated using their XRD patterns and the Debye–Scherrer equation (Figure 7). In this way, the sizes of 64 nm for Cu-MOF and 73 nm for penicillinoate@Cu-MOF were calculated. Peaks [111], [200], [220], [311], and [222] were observed in the XRD patterns of the synthesized Cu-MOF and penicillinoate@Cu-MOF. According to past studies, these peaks are related to the JCPDS 89-2530 and octahedral crystal structures of copper nanoparticles (Xu et al., 2006; Betancourt-Galindo et al., 2014; Gan et al., 2015; Elango et al., 2018; Li et al., 2018). Based on the obtained results, the observed peaks are well-integrated with the related structures, and no impurities are observed in the final products, which can be caused by the efficient synthesis of the final structures.

**FIGURE 8:**
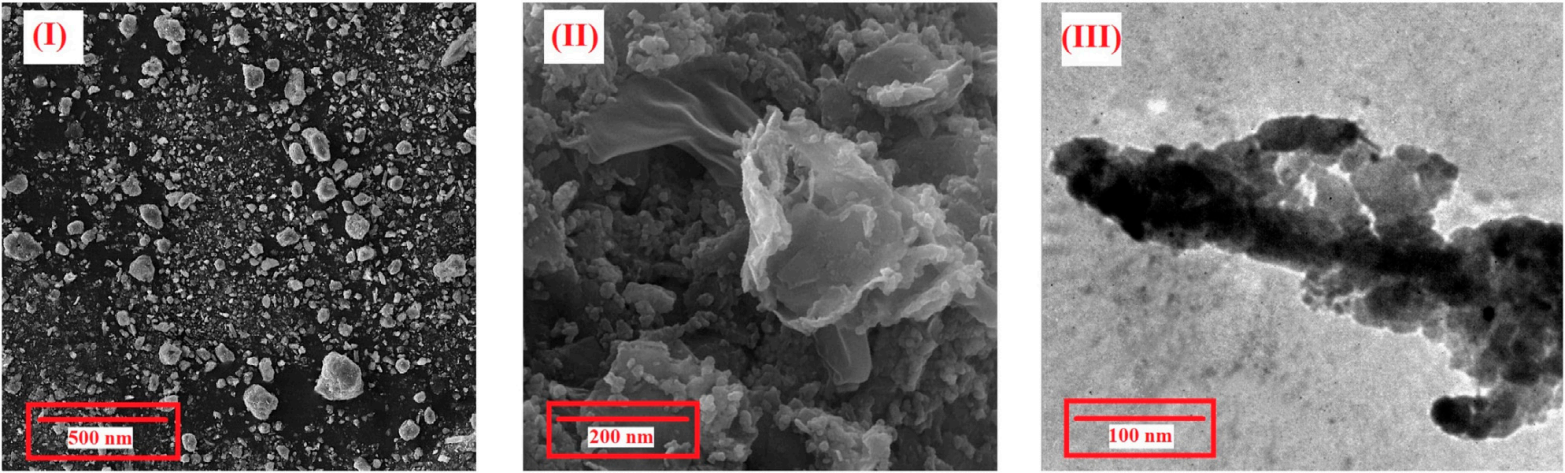
SEM images of Cu-MOF **(I)** and penicillinoate@Cu-MOF **(II)** and TEM images of penicillinoate@Cu-MOF **(III)**.

In addition to the XRD patterns, the nanosize and same morphology of Cu-MOF and penicillinoate@Cu-MOF, which is an important factor in nanostructure and depends on the synthesis method, were proved using the SEM and TEM images (Figure 8). According to the SEM image of Cu-MOF, the sample has a homogeneous morphology without any evidence of the agglomeration process. The SEM image of penicillinoate@Cu-MOF confirms the formation process of Cu-MOF nuclei in the final structure. The TEM image of penicillinoate@Cu-MOF confirmed a homogeneous and uniform morphology of the products. As an important result, the surface stability of compounds can be attributed to the efficient microwave radiation developed in this study.

**FIGURE 9:**
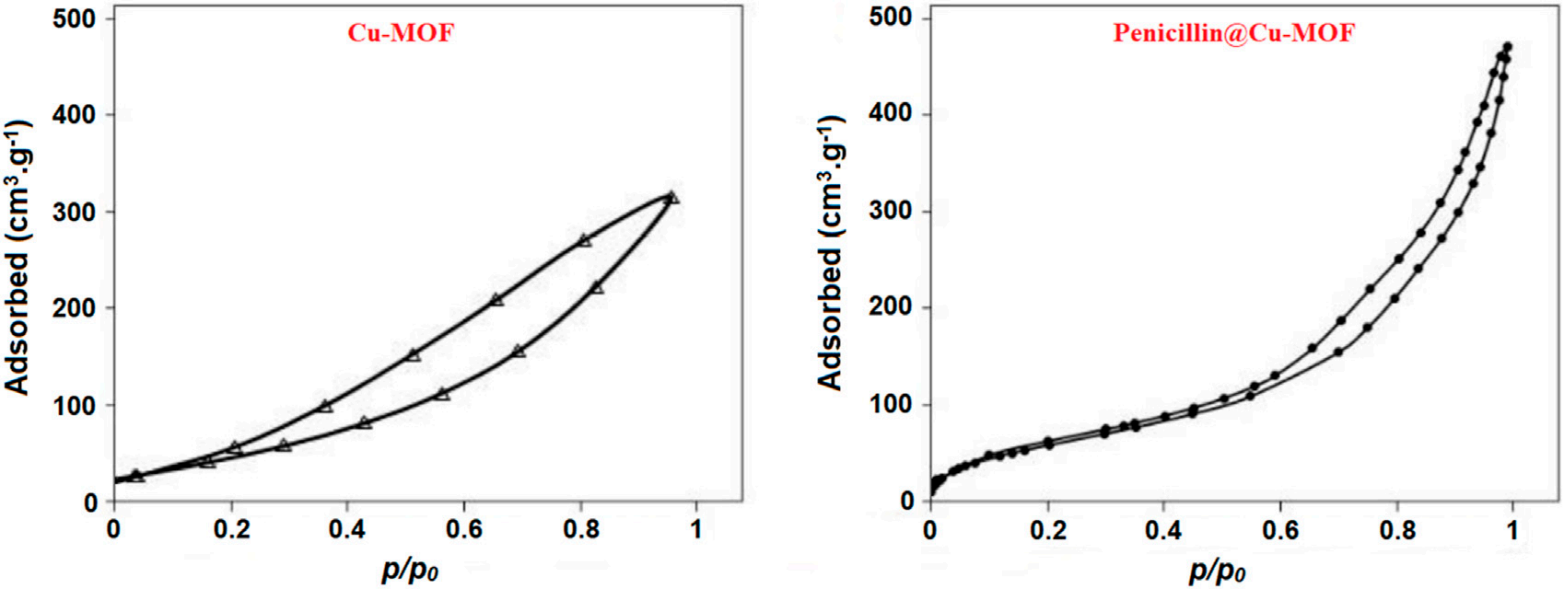
Nitrogen adsorption/desorption curves of Cu-MOF and penicillinoate@Cu-MOF.

The specific active area, which is another characteristic of nanostructures that contributes to their unique properties and activities, is another parameter that depends on the synthesis method of these compounds. The specific active areas of Cu-MOF and penicillinoate@Cu-MOF were 27 m^2^/g and 34 m^2^/g, respectively (Figure 9), obtained using their nitrogen absorption and desorption curves. From the obtained results of specific active areas, it can be claimed that the microwave radiation method is a suitable method for the synthesis of the desired products.

**FIGURE 10:**
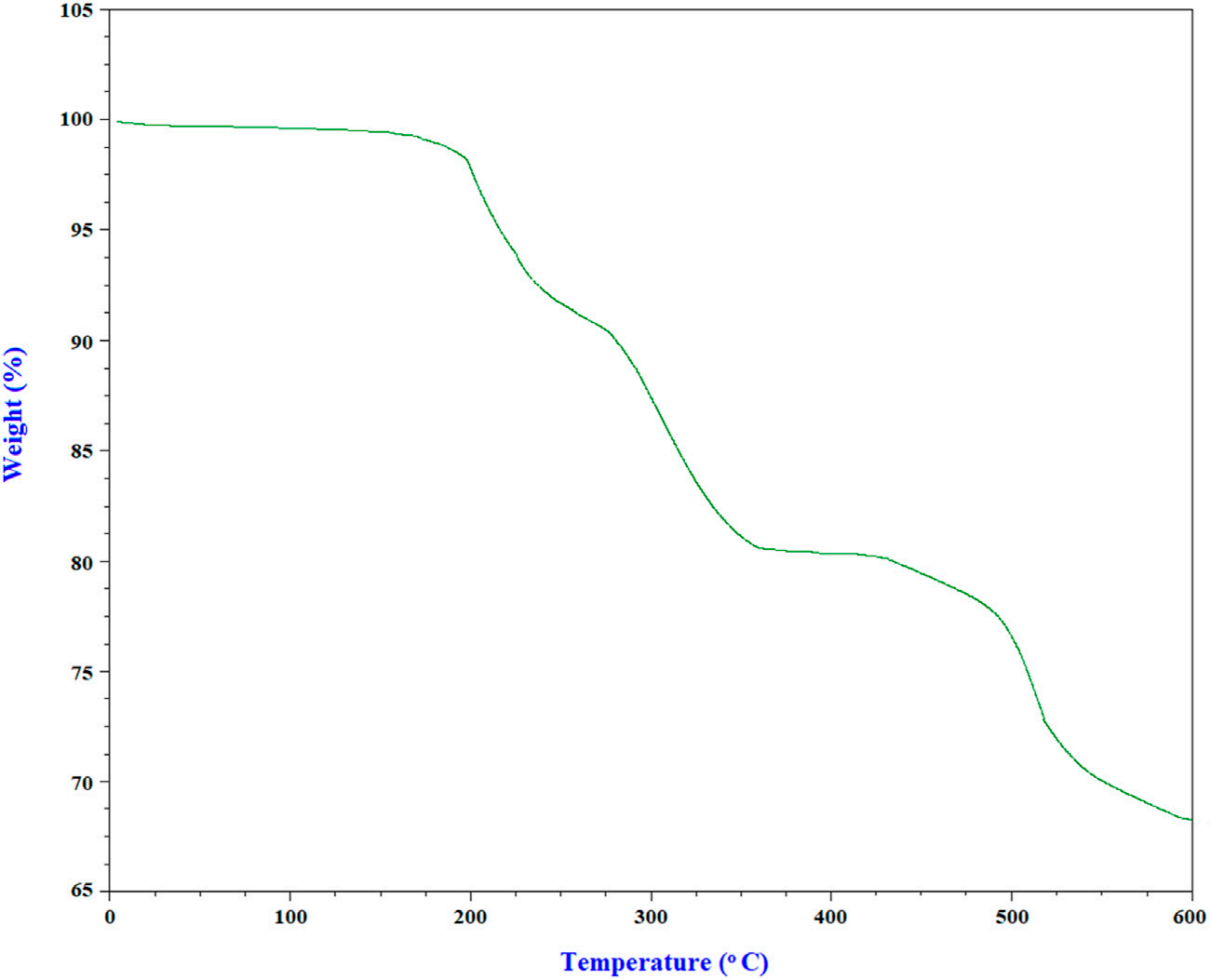
TGA curve of penicillinoate@Cu-MOF

The weight loss obtained from TGA related to penicillinoate@Cu-MOF is shown in Figure 10. Therefore, it can be stated that the initial weight loss is due to the water molecules absorbed on the surface of structures and the solvent molecules trapped in the pores of the penicillinoate@Cu-MOF sample. The next weight loss, at a temperature below 275 °C, can be related to the decomposition of penicillin molecules. The subsequent weight loss, which is at 275 °C–350 °C, can be attributed to the organic groups that bind to the Cu-MOF. Finally, the last weight loss, which is at 425 C–525 C, can be attributed to breaking of the complex structure of the product. The important thing to mention here is the complete stability of the product up to a temperature of 200 C, which is significant in biological applications. This behavior can be related to the selection of optimal MOF nanostructures and the development of efficient microwave irradiations.

### 3.2 Results of antibacterial tests on penicillin and penicillinoate@Cu-MOF

The antibacterial properties of penicillin, Cu-MOF, and penicillinoate@Cu-MOF were investigated on bacterial strains such as *Rhodococcus equi* (ATCC 25729), *Bacillus cereus* (ATCC 11778), *Staphylococcus aureus* (ATCC 29213), *Staphylococcus epidermidis* (ATCC 14990), *Streptococcus equinus* (ATCC 15352), *Streptococcus agalactiae* (ATCC 12386), *Yersinia enterocolitica* (ATCC 9610), *Escherichia coli* (ATCC 25922), *Pseudomonas aeruginosa* (ATCC 15442), *Proteus mirabilis* (ATCC 7002), *Proteus vulgaris* (ATCC 6380), and *Salmonella enterica subsp. enterica* (ATCC 14028).

**FIGURE 11:**
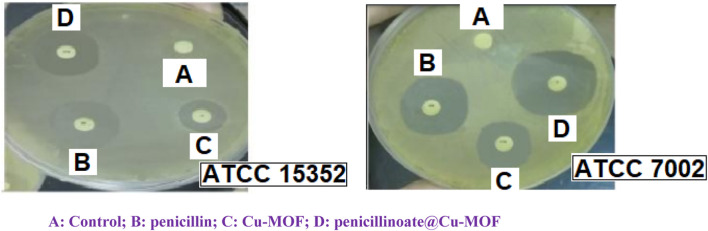
Example of IZA in antibacterial activity. **(A)** Control; **(B)** penicillin; **(C)** Cu-MOF; (**D)** penicillinoate@Cu-MOF

In the tests, as mentioned in the Test method section, IZD, MIC, and MBC were measured (Table 2; Figure 11).

**TABLE 2:** Antibacterial activity of analysis of penicillin, Cu-MOF, and penicillinoate@Cu-MOF (mean, n = 3).

Strain		Parameters	Compound		
			Penicillin	Cu-MOF	Penicillinoate@Cu-MOF
Gram-positive	ATCC 25729	MIC (μg/mL)	8	-	8
		MBC (μg/mL)	16	-	8
		IZD (mm)	19.46	-	18.30
	ATCC 11778	MIC (μg/mL)	-	32	8
		MBC (μg/mL)	-	32	16
		IZD (mm)	-	15.21	19.09
	ATCC 29213	MIC (μg/mL)	16	32	8
		MBC (μg/mL)	32	64	16
		IZD (mm)	17.34	18.40	18.97
	ATCC 14990	MIC (μg/mL)	8	-	8
		MBC (μg/mL)	16	-	16
		IZD (mm)	16.34	-	17.43
	ATCC 15352	MIC (μg/mL)	16	32	16
		MBC (μg/mL)	32	64	16
		IZD (mm)	19.93	17.52	20.43
	ATCC 12386	MIC (μg/mL)	-	64	32
		MBC (μg/mL)	-	128	16
		IZD (mm)	-	14.91	17.62
Gram-negative	ATCC 9610	MIC (μg/mL)	-	8	8
		MBC (μg/mL)	-	16	8
		IZD (mm)	-	16.77	15.46
	ATCC 25922	MIC (μg/mL)	-	32	32
		MBC (μg/mL)	-	64	64
		IZD (mm)	-	17.79	18.01
	ATCC 15442	MIC (μg/mL)	-	64	64
		MBC (μg/mL)	-	128	64
		IZD (mm)	-	14.37	15.29
	ATCC 7002	MIC (μg/mL)	16	32	8
		MBC (μg/mL)	32	64	16
		IZD (mm)	18.31	15.51	22.62
	ATCC 6380	MIC (μg/mL)	-	32	16
		MBC (μg/mL)	-	64	32
		IZD (mm)	-	12.81	14.95
	ATCC 14028	MIC (μg/mL)	8	-	2
		MBC (μg/mL)	16	-	4
		IZD (mm)	19.58	-	18.23

*Rhodococcus equi* (ATCC, 25729), *Bacillus cereus* (ATCC, 11778), *Staphylococcus aureus* (ATCC, 29213), *Staphylococcus epidermidis* (ATCC, 14990), *Streptococcus equinus* (ATCC, 15352), *Streptococcus agalactiae* (ATCC, 12386), *Yersinia enterocolitica* (ATCC, 9610), *Escherichia coli* (ATCC, 25922), *Pseudomonas aeruginosa* (ATCC, 15442), *Proteus mirabilis* (ATCC, 7002), *Proteus vulgaris* (ATCC, 6380), and *Salmonella enterica subsp. enterica* (ATCC, 14028).

Penicillin is an antibiotic that is effective against some bacterial pathogens. However, as shown in Table 2, it is ineffective on some strains such as *B. cereus, S. agalactiae*, *Y. enterocolitica*, *E. coli*, *P. aeruginosa*, and *P. vulgaris*. The results given in the table show that Cu-MOF is effective on them. The results show that Cu-MOF is ineffective on some strains, but penicillin is effective. Therefore, the final synthesized product (penicillinoate@Cu-MOF) containing penicillin and MOF was effective against a broader range of bacterial strains. The effectiveness of Cu-MOF can be attributed to the presence of copper with antibacterial activity. As seen in the table, the final product containing penicillin and Cu-MOF was more effective on some strains than penicillin and on some strains than Cu-MOF. This case can be attributed to the placement of penicillin in the nanostructure substrate and also to the higher specific active surface of the final product compared to others. (Gwon et al., 2020; Liu et al., 2021; Asgari et al., 2022; He et al., 2022).

### 3.3 Results of antioxidant tests on penicillin, Cu-MOF, and penicillinoate@Cu-MOF

In the investigation of the antioxidant activity, the antioxidant activity of penicillin, Cu-MOF, and penicillinoate@Cu-MOF were investigated.

DPPH free radical inhibition % was calculated using the following formula.

$$\text{Inhibition }\%=\left[\left(\text{Absorbance of DPPH }–\text{ Absorbance of subfilter solution}\right)/\;\left(\text{Absorbance of DPPH}\right)\right]\times 100.$$ (1)

Eq.1 determines the percentage inhibition of DPPH free radicals.

In addition to the inhibition %, the IC_50_ value was calculated, and the results are shown in Table 3.

**TABLE 3:** Antioxidant activity of analysis of penicillin, Cu-MOF, and penicillinoate@Cu-MOF (mean, n = 3).

	Concentration (μg/mL)	Compound		
		Penicillin	Cu-MOF	Penicillinoate@Cu-MOF
Inhibition %	5	2	8	12
	10	5	17	26
	15	11	25	37
	20	14	38	45
IC_50_ (μg/mL)		68.92	26.34	21.30

In the assessment of antioxidant activity, IC_50_ values for penicillin, Cu-MOF, and penicillinoate@Cu-MOF were determined as 68.92 μg/mL, 26.34 μg/mL, and 21.30 μg/mL, respectively. Based on these results, it was determined that the antioxidant activity of penicillinoate@Cu-MOF is more than that of penicillin and Cu-MOF. The high oxidizing activity of penicillinoate@Cu-MOF can be attributed to the absorption of DPPH molecules in the cavities of the final product (Ke et al., 2019; AbouAitah et al., 2021).

## 4 Conclusion

In this study, a new nanostructure containing penicillin and Cu-MOF was synthesized, and the desired and proposed product containing penicillin was confirmed by FT-IR spectrums, EDAX analysis, elemental analysis, XRD patterns, SEM images, nitrogen adsorption/desorption curves, and TGA curves. The size of the final synthesized product was 74 nm. The same morphology, specific surface area of 34 m^2^/g, and thermal stability of up to 200°C were among the unique physical and chemical characteristics of the synthesized product. The antibacterial activity of the synthesized product was tested against 12 pathogenic bacterial strains, and the IZD, MIC, and MBC were measured and reported. Interesting results were observed from the antibacterial tests. For example, the final product was effective on strains for which penicillin and Cu-MOF were not effective alone. In addition, a higher effectiveness was observed compared to the strains for which which penicillin and Cu-MOF were effective. The antioxidant activity of the synthesized product was tested using the DPPH method and % inhibition, and the IC_50_ values were reported. These remarkable features of the newly synthesized compound can be attributed to its nano-sized, high specific surface area, which has a direct relationship with the synthesis method, microwave method. Therefore, as a final result, it can be stated that nanostructures with higher biological properties can be synthesized using the microwave method and combining bioactive materials.

## Data Availability

The original contributions presented in the study are included in the article/Supplementary materials; further inquiries can be directed to the corresponding author.
